# Transition from Child and Adolescent to Adult Mental Health Services in Young People with Depression: On What Do Clinicians Base their Recommendation?

**DOI:** 10.1155/2023/8495521

**Published:** 2023-10-31

**Authors:** Larissa S. van Bodegom, Mathilde M. Overbeek, Suzanne E. Gerritsen, Athanasios Maras, Manon H. J. Hillegers, Dieter Wolke, Dimitris Rizopoulos, Giovanni Allibrio, Therese A. M. J. van Amelsvoort, Rebecca Appleton, Marco Armando, Tomislav Franić, Giovanni de Girolamo, Jason Madan, Lidia Manenti, Francesco Margari, Fiona McNicholas, Adriana Pastore, Moli Paul, Diane Purper-Ouakil, Francesco Rinaldi, Melanie C. Saam, Paramala J. Santosh, Anne Sartor, Ulrike M. E. Schulze, Giulia Signorini, Swaran P. Singh, Cathy Street, Priya Tah, Elena Tanase, Sabine Tremmery, Helena Tuomainen, Gwendolyn C. Dieleman

**Affiliations:** ^1^Yulius Mental Health Organization, Yulius Academy, Dordrecht, 3300 BA, Netherlands; ^2^Department of Child and Adolescent Psychiatry and Psychology, Erasmus Medical Center, Rotterdam, 3000 CB, Netherlands; ^3^ARQ National Pyschotrauma Centre, Diemen, Netherlands; ^4^Clinical Child and Family Studies, Vrije Universiteit Amsterdam, Amsterdam, 1081 BT, Netherlands; ^5^Department of Psychology, University of Warwick, Coventry CV4 7AL, UK; ^6^Department of Biostatistics, Erasmus Medical Center, Rotterdam, 3000 CB, Netherlands; ^7^Department of Epidemiology, Erasmus Medical Center, Rotterdam, 3000 CB, Netherlands; ^8^Child And Adolescent Neuropsychiatric Service, ASST Spedali Civili, Brescia, Italy; ^9^Department of Psychiatry and Neuropsychology, University of Maastricht, Maastricht, Netherlands; ^10^Mondriaan Mental Health Care, Heerlen, Netherlands; ^11^NIHR Mental Health Policy Research Unit, Division of Psychiatry, University College London, London W1T 7NF, UK; ^12^University Service for Child and Adolescent Psychiatry, Department of Psychiatry, Centre Hospitalier Universitaire Vaudois, University of Lausanne, Lausanne, Switzerland; ^13^Department of Psychiatry, University Hospital Split, Split 21000, Croatia; ^14^School of Medicine, University of Split, Split 21000, Croatia; ^15^Psychiatric Epidemiology and Evaluation Unit, IRCCS Istituto Centro San Giovanni di Dio Fatebenefratelli, Brescia 25125, Italy; ^16^Warwick Medical School, University of Warwick, Coventry CV4 7AL, UK; ^17^Child Neuropsychiatric Unit, University of Bari, Bari 70121, Italy; ^18^School of Medicine & Medical Science, University College Dublin, Dublin D04 V1W8, Ireland; ^19^Lucena CAMHS, SJOG, Dublin, Ireland; ^20^Unit of Adolescent Psychiatric Emergency, “Policlinico” Hospital, Bari 70121, Italy; ^21^Centre Hospitalier Universitaire de Montpellier, Saint Eloi Hospital, Montpellier 34090, France; ^22^CESP U1018, PsyDev, University Paris Saclay, UVSQ, INSERM, Versailles, France; ^23^Child And Adolescent Neuropsychiatric Service, ASST of Valcamonica, Esine, Italy; ^24^Department of Child and Adolescent Psychiatry/Psychotherapy, University of Ulm, Ulm 89075, Germany; ^25^Department of Child & Adolescent Psychiatry, Institute of Psychiatry, Psychology and Neuroscience, Kings College London, London SE5 8AF, UK; ^26^Centre for Interventional Paediatric Psychopharmacology and Rare Diseases, South London and Maudsley NHS Foundation Trust, London SE5 8AZ, UK; ^27^HealthTracker Ltd., Kent ME7 1AY, UK; ^28^Klinik für Kinder- und Jugendpsychiatrie und Psychotherapie, Josefinum Augsburg, Augsburg 86154, Germany; ^29^Rees Centre, Department of Education, University of Oxford, Oxford OX2 6PY, UK; ^30^Department of Neurosciences, KU Leuven, Leuven BE-3000, Belgium

## Abstract

**Background:**

Clinicians in Child and Adolescent Mental Healthcare Services (CAMHS) face the challenge to determine who is at risk of persistence of depressive problems into adulthood and requires continued treatment after reaching the CAMHS upper age limit of care-provision. We assessed whether risk factors for persistence were related to CAMHS clinicians' transition recommendations.

**Methods:**

Within the wider MILESTONE cohort study, 203 CAMHS users were classified with unipolar depressive disorder by their clinician, and 185 reported clinical levels of depressive problems on the DSM-oriented Depressive Problems scale of the Achenbach Youth Self Report. Logistic regression models were fitted to both subsamples to assess the relationship between clinicians' transition recommendations and risk factors for persistent depression.

**Results:**

Only clinician-rated severity of psychopathology was related to a recommendation to continue treatment for those classified with unipolar depressive disorder (*N* = 203; OR = 1.45, 95% CI (1.03–2.03), *p* = .044) and for those with self-reported depressive problems on the Achenbach DSM-oriented Depressive Problems scale (*N* = 185; OR = 1.62, 95% CI (1.12–2.34), *p* = .012).

**Conclusion:**

Transition recommendations and need for continued treatment are based on clinical expertise, rather than self-reported problems and needs.

## 1. Introduction

Depressive disorders account for the highest burden of disease among young people in the age range of 10-24 years in high-income countries [1]. Young people with a depressive disorder have a two- to threefold higher risk for a depressive disorder in adulthood [2, 3]. For almost half of young people with depressive disorder (40%), the disorder continues into adulthood following a recurrent pattern, while for 18% of young people, it remains unremitted into adulthood with poor distal outcomes [4, 5]. Overall, depressive disorders are responsible for an 8.6% loss in disability-adjusted life years and have a major impact on society: they reduce the quality of life, affect peer and family relationships, cause absenteeism, and affect performance at work [6–8]. Effective mental healthcare can reduce the burden of disease by 30-40% [9]. Hence, adolescent patients who are at risk of persistence of the depressive disorder may benefit from continued treatment as they become adults [8, 10].

However, many mental health services for children and adolescents (CAMHS) restrict the provision of help to a specific age range, usually somewhere between the ages of 16 and 19 years. This restriction causes a divide between CAMHS and Adult Mental Healthcare Services (AMHS) which may be a barrier to continued treatment. This divide presents CAMHS clinicians with the difficult task of identifying which patients are at risk of persistence of the depressive disorder into adulthood and need continued treatment, and which patients can safely be discharged. Besides young people classified with a depressive disorder, CAMHS users classified with other disorders might also be at risk of depressive symptoms. Especially in clinical samples, as depressive problems are highly comorbid with, for example, anxiety and substance use disorders [7, 11] and are associated with an increased burden of disease [6, 12]. It is therefore important for clinicians to decide both for young people with depressive disorders and for those with depressive symptoms who might be at risk of persistent depression and may need continued treatment.

Increased knowledge of factors associated with persistence of depression could guide clinicians in identifying those at risk and inform their associated transition recommendations. In addition to an onset of depressive disorder before the age of 21 years, the following factors have been consistently associated with persistence of the depressive disorders into adulthood: having peer and family relationship problems, parental psychopathology, more severe impairment, and more severe mental health problems including higher rates of suicidality and comorbid psychiatric disorders (specifically anxiety, personality, and substance use disorders) [6, 10, 13, 14]. From now on, we refer to these risk factors as risk factors for “persistent depression”—which also includes persistence in terms of recurring episodes. However, no studies to date have investigated how risk factors for persistent depression are considered in clinicians' advice about continued treatment for depressed young people facing the upper age limit of their CAMHS.

Although several empirical and longitudinal studies on transition have been conducted [15–21], the MILESTONE cohort study [22] is the first European longitudinal prospective study investigating the transition process in a cohort of CAMHS users with different types of psychopathology. We previously showed that, within the MILESTONE cohort, the CAMHS clinician's recommendation to continue treatment within a mental healthcare setting was primarily determined by clinician-reported severity of psychopathology and self- and parent-reported need for continued treatment. This is in line with transition guidelines such as NICE, which ask clinicians to consider patient preference and self-reported need in their transition recommendation [23]. The recommendation to continue treatment in AMHS rather than CAMHS was mainly associated with treatment- and service-use-related factors, such as length of CAMHS use and availability of appropriate AMHS [24].

The current study investigates whether risk factors for persistent depression are considered in clinicians' advice about continued treatment for depressed young people facing the upper age limit of their CAMHS. Therefore, we focus on two subsamples of young people within the wider MILESTONE cohort: those classified with unipolar depressive disorder by their clinician and those who reported clinical levels of depressive problems on the DSM-oriented Depressive Problems scale of the Achenbach Youth Self Report. We hypothesize that known risk factors for persistent depression into adulthood, in addition to more general factors previously associated with the clinician's transition recommendation—such as self-reported need—are considered in the clinician's advice about future treatment for young people with depression.

## 2. Materials and Methods

### 2.1. Study Design and Participants

The current study is part of the MILESTONE cohort study, a prospective cohort study investigating longitudinal outcomes in a cohort of CAMHS users from 39 CAMHS in Europe (Belgium, Croatia, France, Germany, Ireland, Italy, the Netherlands, and the United Kingdom). For a CAMHS to be eligible, it had to provide medical and psychosocial interventions for children and adolescents with mental health problems and disorders and/or neuropsychiatric/developmental disorders; to be community-based or provide outpatient or inpatient care; to be publicly or privately funded; to have a formal upper age limit for providing care to young people; and to be responsible for the transition to adult service [22]. Signed site agreements were obtained from participating sites prior to the start of the study. Databases of participating CAMHS were scrutinised by CAMHS personnel to identify young people approaching the upper age limit of their CAMHS and meeting the inclusion criteria. For young people to be eligible, they had to receive treatment and approach the upper age limit of their CAMHS; they had to be within one year before or three months after the upper age limit; they had a minimum IQ of 70 or no indication of intellectual impairment; and they were expected to be able to complete questionnaires. CAMHS clinicians were asked to introduce the study and to seek consent to be contacted by a research assistant for further information. According to national laws and medical ethical committee regulations, country-specific consent procedures were followed for young people, parents and clinicians. If young people consented to participate, a parent, and a clinician (a mental health professional responsible for the care of the young person) were invited to participate in the study as well. In total, 763 young people, 651 of their parents, and 699 of their clinicians completed the baseline assessment after providing written consent. Supplementary Figure S1 describes the flow of participants in the eligibility, recruitment, and follow-up process. Due to local privacy laws, only limited information could be collected during the eligibility and recruitment procedure. A more detailed description of the recruitment procedures, research aims, measurements, and the cohort is presented in earlier papers [22, 25]. The UK National Research Ethics Service Committee West Midlands–South Birmingham (15/WM/0052) and ethics boards in participating countries approved the study protocol (ISRCTN83240263; NCT03013595.

For the current study, we identified two partially overlapping samples of young people with clinical depression within the wider MILESTONE cohort study (Figure S1). No further data was collected, and data was used to study the transition from CAMHS to AMHS, for which all participants provided their written consent. Our first sample (*N* = 203; 72.9% female and 77.8% White) consists of young people in CAMHS who were classified with unipolar depressive disorder by their clinician (see Table S1 for a specification of the clinical classification category “unipolar depressive disorder”). A second sample (*N* = 185; 76.8% female and 77.2% White) consists of young people with self-reported clinical levels of depressive problems over the past six months as assessed with the DSM-oriented Depressive Problems scale of the Youth Self Report (<18; YSR; [26]) or the Adult Self Report (>18; ASR; [27]). Raw scores were converted into *t*-scores according to the manual and used to identify those with self-reported depressive problems in the clinical range (*t* ≥ 70).

### 2.2. Procedure

After consent was obtained, young people and their parents were invited to the CAMHS clinic for a two-hour assessment, preferably six months before reaching the upper age limit. We conducted interviews with young people, their parents, and their clinicians separately to gather sociodemographic information and information on need for care based on the Health of the Nation Outcome Scales for Children and Adolescents (HoNOSCA; [28]). Additionally, online questionnaires were completed by young people, parents, and clinicians at the HealthTracker™ platform, which could be completed at home if necessary. Clinical information was provided by the clinician or by accessing medical files.

### 2.3. Measures

We describe our main constructs (clinical depression and transition recommendations) in detail below and list studied factors associated with a transition recommendation, risk factors for persistent depression, and covariates briefly. Further details are presented in Table S2.

#### 2.3.1. Clinical Depression

*(1) Clinical Classifications*. Classifications registered in the medical records were provided by CAMHS clinicians and were based on the Diagnostic and Statistical Manual of Mental Disorders, version IV or 5, or the International Classification of Diseases, version 10.

*(2) Self-Reported Depressive Problems*. Problems over the past six months were assessed with the DSM-oriented Depressive Problems scale of the YSR and ASR, which have been used extensively in different contexts and have shown excellent psychometric properties. Raw scores were converted into *t*-scores according to the manual and used to differentiate between normal (*t* ≤ 64), borderline clinical (*t* = 65–69), and clinical (*t* ≥ 70) self-reported depressive problems.

#### 2.3.2. Transition Recommendation at Baseline

At baseline, clinicians indicated on the Transition Readiness and Appropriate Measure (TRAM; [29]) what type of care they considered most appropriate for the young person: “be discharged (1),” “treated by GP/family doctor (2),” “treated by other mental health services (specify) (3),” “remain with their current service (4),” or “transition to AMHS (5)”. We created a dichotomous variable to distinguish between a recommendation for ‘continuity' of treatment within a mental healthcare setting (3, 4, or 5) and “discontinuity” (1 or 2). A second dichotomous variable was created for those recommended to continue their treatment to distinguish between a “CAMHS recommendation” and an “AMHS recommendation.”

#### 2.3.3. General Factors Associated with a Recommendation to Continue Treatment

The following factors, which have been associated with the clinicians' transition recommendation in a previous study within this cohort [24] were included clinician-rated severity of psychopathology (Clinical Global Impression–Severity scale (CGI-S; [30])), psychotic experiences (Development and Well-Being Assessment (DAWBA; [31])), everyday functional skills (Specific Levels of Functioning (SLOF; [32])), psychotropic medication use (Service Receipt Inventory EU version (CSSRI-EU; [33])), length of CAMHS use (sociodemographic interview), the availability of appropriate AMHS according to the clinician (TRAM) and self- and parent-reported need for continued treatment (TRAM).

#### 2.3.4. Risk Factors for Persistent Depression

The following risk factors for persistent depression, identified in the literature [10, 13], were included suicidal thoughts and behaviours (TRAM), history of attempted suicide (sociodemographic interview), self-reported family dysfunction (HoNOSCA), self-reported peer relationship problems (HoNOSCA), bullying (adapted from the Retrospective Bullying and Friendship Interview Schedule ([34]), psychopathology in biological parents (sociodemographic interview), somatic comorbidity (sociodemographic interview), and psychiatric comorbidity (based on clinical classifications).

#### 2.3.5. Covariates

Sociodemographic information used as covariates included gender, highest level of parental education (sociodemographic interview), and country.

### 2.4. Statistical Analysis

First, we assessed which factors were associated with the clinician's transition recommendation to continue treatment for young people classified with unipolar depressive disorder, by fitting three penalized logistic regression models in which the clinician's recommendation was included as the dependent variable. We fitted (1) a covariate-only model, (2) a model with general factors associated with a transition recommendation as independent variables, and (3) a model with general factors associated with a transition recommendation and risk factors for persistent depression as independent variables. The fit of these models was compared using likelihood ratio tests. As a check, we built a fourth model in which we excluded clinician-rated severity of psychopathology as an independent variable, as it is a strong general index for psychopathology and one of the main risk factors associated with persistent depression, and therefore might suppress other associations in our multivariate models. Secondly, we fitted similar models to assess which factors were associated with being recommended to continue treatment in AMHS (rather than CAMHS) for young people classified with unipolar depressive disorder. Lastly, all analyses were repeated in the sample of young people with self-reported depressive problems in the clinical range.

All statistical analyses were performed using R Statistics for Windows, with a significance level of *α* = 0.05. Gender, country, and parents' highest completed level of education were added as covariates to account for potential confounding. Penalized logistic regression models were fitted using the “arm” package [35], due to separation problems caused by the covariate “country.” Assumptions were tested with the “DHARMa” package [36]. Multicollinearity was not present as indicated by a maximum squared adjusted generalized variance inflation factor (GVIF^(1/(2^∗^Df)^), comparable to VIF) under 2. As we incorporated a large amount of independent variables, we inspected the calibration of all models using the “rms” package [37]. We only interpreted associations when models had sufficient predictive accuracy, as indicated by a corrected c-statistic above 0.70.

#### 2.4.1. Missing Data and Multiple Imputations

Within our subsamples, data on transition recommendations were missing for 20-21.2% of young people. The missing data on studied factors ranged from 0% up to 38.4% for psychopathology in biological parents (Table 1). We assumed data was missing at random. To account for missing data, we applied multiple imputation to all variables included in the analyses before models were fitted using “mice” [38] and “miceadds” [39]. We reported pooled odds ratios and corresponding 95% confidence intervals based on 30 imputed datasets. The sample was described based on original nonimputed data.

## 3. Results

### 3.1. Sample Description

Within the original MILESTONE cohort, we identified two samples of young people with clinical depression: (1) young people classified with unipolar depressive disorder (*N* = 203), and (2) young people with self-reported depressive problems in the clinical range (*N* = 185). For each sample, transition recommendations and descriptives are presented in Table 1. Proportions of clinical classifications for young people with self-reported depressive problems are presented in Figure S2.

### 3.2. Factors Associated with the Clinicians' Recommendation to Continue Treatment

Table 2 shows which factors are associated with the clinicians' recommendation to continue treatment for young people classified with unipolar depressive disorder. Corrected c-statistics above .70 showed that all models had sufficient predictive accuracy to interpret associations. The model with general factors predicted the “continuity recommendation” significantly better than the covariate-only model (*p* = .008). However, adding risk factors for persistent depression did not further improve the model fit (*p* = .855). For young people with unipolar depressive disorder, only more severe clinician-rated psychopathology predicted a recommendation to continue treatment (OR = 1.45, 95% CI (1.03–2.03), *p* = .044). After excluding clinician-rated severity of psychopathology as an independent variable from the model, the model did not predict a “continuity recommendation” significantly better than the covariate-only model (*p* = .182).

For young people with self-reported depressive problems in the clinical range, similar results were found (Table S3). More severe clinician-rated psychopathology increased the odds of a recommendation to continue treatment (OR =1.62, 95% CI (1.12–2.34), *p* = .012), while other general factors and risk factors for persistent depression were not related to a “continuity recommendation” for young people with self-reported depressive problems either. Neither predicted the model a “continuity recommendation” significantly better than the covariate only model, after excluding clinician-rated severity of psychopathology as an independent variable (*p* = .537).

### 3.3. Factors Associated with an AMHS-Recommendation at Baseline

Table S4 shows which factors are associated with a recommendation to continue treatment in AMHS for young people with unipolar depressive disorder. Even though the groups were small (*N* = 39 were recommended to transition to AMHS), corrected c-statistics above .70 showed that all models had sufficient predictive accuracy to interpret associations. The model with general factors did not predict an “AMHS recommendation” significantly better than the covariate-only model (*p* = .367), nor did adding risk factors for persistent depression improve the model fit (*p* = .961). For young people with self-reported depressive problems in the clinical range, the model with general factors also did not predict an “AMHS recommendation” significantly better than the covariate-only model (*p* = .175), nor did adding risk factors for persistent depression improve the model fit (*p* = .933) (Table S5).

## 4. Discussion

The current study investigated CAMHS clinicians' transition recommendations among young people classified with unipolar depressive disorder and young people with self-reported clinical depressive problems. We examined whether known risk factors for persistent depression into adulthood and more general factors previously associated with this recommendation are considered in clinicians' transition recommendations. We found that only clinician-rated severity of psychopathology was associated with the clinicians' recommendation to continue treatment, and—contrary to the NICE transition guidelines—self-reported need for continued treatment does not seem to influence this recommendation. Furthermore, none of the risk factors for persistent depression and factors associated with a transition recommendation in general were related to a recommendation to continue treatment in AMHS rather than in CAMHS.

Previous findings from the MILESTONE cohort [24] showed the CAMHS clinician's recommendation to continue treatment within a mental healthcare setting was primarily determined by clinician-reported severity of psychopathology and self- and parent-reported need for continued treatment. The recommendation to continue treatment in AMHS rather than CAMHS was mainly associated with treatment and service-use-related factors, such as length of CAMHS use and availability of appropriate AMHS [24]. Surprisingly, the clinician's recommendation to continue treatment within the subgroup of young people with clinical depression, currently under investigation, was only determined by clinician-reported severity of psychopathology. Even though it seems appropriate that the clinician's recommendation to continue treatment is based on the clinical assessment of severity of psychopathology, the question is whether current self-reported problems and need for continued treatment are reflected in this assessment. In other words, are clinicians sufficiently aware of young people's current needs? Our study showed that young people with lower clinician-rated severity of psychopathology were less likely to be recommended to continue treatment, while they also reported relatively high levels of self-reported problems and a need for continued treatment. Furthermore, half of young people with self-reported clinical depressive problems were not classified with a depressive disorder. This may indicate that they did not receive appropriate treatment for their current mental health problems, while they did report a high need for continued treatment and had high scores on risk factors for persistent depression. The fact that, in the current study, self- and parent-reported need for continued treatment was not found to be associated with the clinician's recommendation to continue treatment could be explained by limited variation in reported need for treatment for an effect to be found. Overall, young people with a depressive disorder or depressive symptoms and their parents more often reported a need for continued treatment than other CAMHS users (i.e., the remaining part of the MILESTONE cohort).

It seems that CAMHS clinicians usually do not include other risk factors for persistent depression in their recommendation to continue treatment besides their self-rated severity of psychopathology. This is unfortunate as self- and parent-reported need for continued treatment and other risk factors should also guide clinicians in their decision about who could benefit from continued treatment in adulthood [10, 23]. The early age of onset of the depression and the presence of parental psychopathology could be used as indicators for discussing the need of a recommendation to continue treatment, as these factors were consistently found to be predictors of chronicity [13]. More importantly, transition recommendations seem to be entirely based on objective need, whether subjective need might be more important and even a key determinant of need for care.

Finally, none of the general factors and risk factors for persistent depression was associated with the recommendation to continue treatment in AMHS rather than in CAMHS. Most young people who were recommended to continue care were recommended to remain in CAMHS. Therefore, integrated mental healthcare might be the solution for young people in need of continued treatment, as a transition would not be necessary [40]. However, due to the small amount of young people being recommended to continue treatment in AMHS, the groups in the current study could also have been too small to detect associations.

### 4.1. Strengths and Limitations

One of the main strengths of the current study is that it studies the largest cohort of transition-age youth across different service structures and settings with extensive quantitative assessments. Therefore, detailed information is available on many constructs relevant to transitional care. Furthermore, we studied clinical depression from two perspectives: based on clinical classifications and based on self-reported depressive problems in the clinical range. However, there are several limitations to the findings of this study as well. First, studying subsamples within the MILESTONE cohort resulted in relatively small samples for studying logistic regression models with several independent variables. However, corrected c-statistics above .70 showed that all models had sufficient predictive accuracy to interpret associations between dependent and independent variables. Secondly, samples were selected based on clinical classifications, rather than using a standardized diagnostic tool. Therefore, the consistency of this sample may be questioned. Nevertheless, these are young people in care at CAMHS whom the clinician has diagnosed with a depression. Another limitation, related to the one above, might be that there is some degree of imprecision in the data provided through medical files, as registers might not always have been updated properly. Fortunately, medical files were only consulted for information that does not change regularly, and in almost all cases, clinicians provided the information themselves. Furthermore, CAMHS participating in MILESTONE were not selected at random but were affiliated with the MILESTONE consortium and their network of mental health organisations. Additionally, a selection bias may have been introduced within the wider MILESTONE cohort by the response rate of 45.1%. However, it is less likely that the generalizability is affected by a potential selection bias, as variables on which a selection could have taken place were included as covariates in the analyses [41]. Lastly, the proportion of missing information on some measures was considerable. However, an analysis of missing data previously conducted [25] supported our assumption that the missingness was related to observed data, therefore missingness was adequately accounted for by multiple imputation [42].

### 4.2. Practical Implications

This study shows that clinician-rated severity of psychopathology was the only factor associated with the recommendation to continue treatment for young people with clinical depression reaching the upper age limit of their CAMHS. Although it seems appropriate that the recommendation to continue treatment is related to a higher severity of psychopathology, it is recommended to also pay attention to the current self-reported problems and needs of young people—as previously recommended by the NICE guidelines. As parents and clinicians seem to interpret the severity of psychopathology and the need for continued treatment differently than young people, and as risk factors for persistent depression seem not to be influencing the transition recommendation, we recommend using a multidimensional view to estimate severity of psychopathology, risk for persistent depression and need for continued treatment prior to transition decision-making—i.e., discussing what are the most important complaints and needs prior to and regarding transition according to young people, their parents, and their clinicians. Being aware of these different perspectives and discussing transition may encourage joint decision-making and improve transition experiences.

## Figures and Tables

**Table 1:** Transition recommendations and descriptives of young people classified with unipolar depressive disorder (*N* = 203) and young people with self-reported depressive problems (*N* = 185).

	Classified with unipolar depressive disorder by CAMHS clinician	Self-reported depressive problems
	*N* = 203	*N* = 185
*Transition recommendation*		
Clinicians' transition recommendation (% (*n*))		
Recommended to discontinue treatment (% (*n*))	20.2 (41)	17.8 (33)
Recommended to continue treatment (% (*n*))	58.6 (119)	62.2 (115)
Recommended to remain in CAMHS	48.7 (58)	46.9 (54)
Recommended to transition to AMHS	32.8 (39)	35.7 (41)
Recommended to be treated by other MHS	18.5 (22)	17.4 (20)
Missing	21.2 (43)	20.0 (37)

*Depression*		
Unipolar depressive disorder classification (% (*n*))	100 (203)	49.7 (92)
Self-reported depressive problems (% (*n*))		
Normal range (*t* − score ≤ 64)	26.6 (54)	—
Subclinical range (*t* − score = 65–69)	14.8 (30)	—
Clinical range (*t* − score ≥ 70)	45.3 (92)	100 (185)
Missing	13.3 (27)	—

*Sociodemographic characteristics*		
Gender: female (% (*n*))	72.9 (148)	76.8 (142)
Highest level PC education (% (*n*))		
Primary/secondary/vocational	40.9 (83)	40.5 (75)
University	23.6 (48)	24.9 (46)
Missing	35.5 (72)	34.6 (64)
Psychopathology in biological parents (% (*n*))		
No psychopathology	33.5 (68)	31.9 (59)
Psychopathology in one or both biological parents	28.1 (57)	30.8 (57)
Missing	38.4 (78)	37.3 (69)

*Severity and impairment*		
Clinician-rated severity of psychopathology (*M* (SD))	3.74 (1.35)	3.95 (1.33)
Suicidal thoughts and behaviours (% (*n*))		
None	47.3 (96)	35.7 (66)
Suicidal thoughts and behaviours	44.8 (91)	64.3 (119)
Missing	7.9 (16)	—
Lifetime suicide attempt (% (*n*))		
No	41.9 (85)	43.8 (81)
Yes	46.8 (95)	50.8 (94)
Missing	11.3 (23)	5.4 (10)
Psychotropic medication use (% (*n*))		
No	26.6 (54)	29.7 (55)
Yes	58.6 (119)	65.9 (122)
Missing	14.8 (30)	4.3 (8)

*Need for continued treatment*		
Self-reported need for continued treatment (% (*n*))		
No	24.1 (49)	16.8 (31)
Yes	68.0 (138)	83.2 (154)
Missing	7.9 (16)	—
Parent-reported need for continued treatment (% (*n*))		
No	9.4 (19)	6.5 (12)
Yes	57.1 (116)	59.5 (110)
Missing	33.5 (68)	34.1 (63)

*Note.* Based on orginial, nonimputed data. PC=parent/carer.

**Table 2:** Factors associated with a continuity recommendation for young people classified with unipolar depressive disorder (descriptives and model summary).

	Characteristics (original non-imputed data)		OR (95% CI) (model summary on imputed data)⁣^∗^	
	Discontinuity recommendation (*n* = 41)	Continuity recommendation (*n* = 119)	Model with general factors	Model expanded with risk factors for persistent depression
*General factors associated with a recommendation to continue treatment*				
Clinician-rated severity of psychopathology (M (SD))	2.17 (1.00)	4.18 (1.10)	**1.45 (1.03–2.03)**	**1.47 (1.03–2.10)**
Psychotic experiences (% (*n*))				
0 or 1 experience(s)	46.3 (19)	53.8 (64)	Ref	Ref
2-16 experiences	31.7 (13)	29.4 (35)	0.51 (0.22–1.19)	0.57 (0.23–1.43)
Missing	22.0 (9)	16.8 (20)		
Everyday functional skills (M (SD))	4.43 (0.43)	4.31 (0.46)	0.61 (0.24–1.53)	0.75 (0.27–2.12)
Self-reported need for continued treatment (% (*n*))				
No	46.3 (19)	22.7 (27)	Ref	Ref
Yes	51.2 (21)	70.6 (84)	1.68 (0.65–4.30)	1.61 (0.60–4.31)
Missing	2.4 (1)	6.7 (8)		
Parent-reported need for continued treatment (% (*n*))				
No	26.8 (11)	5.9 (7)	Ref	Ref
Yes	46.3 (19)	63.9 (76)	2.59 (0.83–8.11)	2.44 (0.74–7.98)
Missing	26.8 (11)	30.3 (36)		

*Risk factors for persistent depression*				
Suicidal thoughts and behaviours (% (*n*))				
None	61.0 (25)	47.1 (56)		Ref
Suicidal thoughts and behaviours	36.6 (15)	46.2 (55)		0.71 (0.31–1.63)
Missing	2.4 (1)	6.7 (8)		
Lifetime suicide attempt (% (*n*))				
No	41.5 (17)	48.7 (58)		Ref
Yes	56.1 (23)	43.7 (52)		0.8 (0.35–1.80)
Missing	2.4 (1)	7.6 (9)		
Peer relationship problems (M (SD))	0.70 (0.91)	1.62 (1.15)		1.25 (0.85–1.82)
Family dysfunction (M (SD))	0.90 (1.02)	1.58 (1.11)		1.00 (0.69–1.44)
Parental psychopathology (% (*n*))				
No psychopathology	34.1 (14)	38.7 (46)		Ref
Psychopathology in one or both biological parents	36.6 (15)	28.6 (34)		1.33 (0.58–3.04)
Missing	29.3 (12)	32.8 (39)		
Victim of bullying (% (*n*))				
No	31.7 (13)	26.9 (32)		Ref
Yes	61.0 (25)	63.0 (75)		1.57 (0.68–3.64)
Missing	7.3 (3)	10.1 (12)		
Somatic comorbidity^1^ (% (*n*))	14.6 (6)	13.4 (16)		1.57 (0.54–4.56)
Psychiatric comorbidity^2^ (% (*n*))	22.0 (9)	34.5 (41)		1.11 (0.48–2.56)
	Optimism Slope		0.28	0.41
	Corrected C-Statistic		0.75	0.74

Note. Penalized logistic regression models were fitted, displaying odds of “continuity recommendation” versus “discontinuity recommendation” as the reference group. Gender, parental education level, and country were added as covariates. ⁣^∗^*n* changes per imputed dataset. YP = young person; PC = parent/carer. ^1^Presence of serious somatic problems such as heart diseases and diabetes; ^2^Presence of classifications of anxiety, personality, and/or substance-use disorders.

## Data Availability

Data may not be shared outside the MILESTONE consortium due to participant consent form restrictions. Examples of instruments used in the study can be made available upon request to the corresponding author.

## Supplementary Materials

Supplementary Materials Figure S1 describes the flow of participants in the process of assessing eligibility, recruitment, and follow-up. Figure S2 presents the proportions of clinical classifications for young people with self-reported depressive problems. See Table S1 for a specification of the clinical classification category “unipolar depressive disorder.” Table S2 presents the details of all measures that are not described in detail in the manuscript: factors associated with a transition recommendation, risk factors for persistent depression, and covariates. Table S3 presents the factors associated with a continuity recommendation for young people with self-reported depressive problems. Factors associated with an AMHS recommendation are presented in Table S4 for young people classified with unipolar depressive disorder and in Table S5 for young people with self-reported depressive problems.
