# Supplementary material for: Transition from Child and Adolescent to Adult Mental Health Services in Young People with Depression: On What Do Clinicians Base their Recommendation?
Source: Depress Anxiety. 2023 Oct 31;2023:8495521. doi: 10.1155/2023/8495521 (PMC11921827; doi:10.1155/2023/8495521)
Supplement: Supplementary Materials — Figure S1 describes the flow of participants in the process of assessing eligibility, recruitment, and follow-up. Figure S2 presents the proportions of clinical classifications for young people with self-reported depressive problems. See Table S1 for a specification of the clinical classification category “unipolar depressive disorder.” Table S2 presents the details of all measures that are not described in detail in the manuscript: factors associated with a transition recommendation, risk factors for persistent depression, and covariates. Table S3 presents the factors associated with a continuity recommendation for young people with self-reported depressive problems. Factors associated with an AMHS recommendation are presented in Table S4 for young people classified with unipolar depressive disorder and in Table S5 for young people with self-reported depressive problems. [file 8495521.f1.docx]

# **SUPPLEMENTARY MATERIAL**

**Table S1**

*Classification of unipolar depressive disorder within MILESTONE*

|  |  | *n* | DSM-5 classifications | ICD-10 classifications |
| --- | --- | --- | --- | --- |
|  |  |  | Included Mood Disorders | Included Mood (Affective) Disorders |
| UNIPOLAR DEPRESSIVE DISORDER | Depressive, Bipolar and Related Disorders / Mood [affective] disorders | 39 | Other Specified Depressive Disorder  Unspecified Depressive Disorder |  |
|  | Major Depressive Disorder, single episode | 82 | Major Depressive Disorder, single episode | F32 Depressive episode |
|  | Major Depressive Disorder, recurrent episode | 55 | Major Depressive Disorder, Recurrent episode | F33 Recurrent depressive disorder |
|  | Dysthymia / Persistent mood (affective) disorder(s) & Disruptive Mood Dysregulation Disorder(s) | 12 | Persistent Depressive Disorder (Dysthymia; + specify ...)  Disruptive Mood Dysregulation Disorder | F34 Persistent mood (affective) disorders |
|  | Other mood (affective) disorders | 9 | - | F38 Other mood (affective) disorders |
|  | Unspecified mood (affective) disorders | 6 | Premenstrual Dysphoric Disorder  Substance/Medication-Induced Depressive Disorder  Depressive Disorder Due to Another Medical Condition | F39 Unspecified mood (affective) disorders |
|  |  |  | Excluded Mood Disorders | Excluded Mood (Affective) Disorders |
| OTHER MOOD DISORDERS | Depressive, Bipolar and Related Disorders / Mood [affective] disorders | 0 |  | F30 Manic Episode |
|  | Bipolar and Related Disorders / Bipolar affective disorder | 16 | Bipolar I Disorder  Bipolar II Disorder  Cyclothymic Disorder  Substance/Medication-Induced Bipolar and Related Disorder  Bipolar and Related Disorder Due to Another Medical Condition  Other Specified Bipolar and Related Disorder  Unspecified Bipolar and Related Disorder | F31 Bipolar Affective Disorder |

*Note.* Due to country differences in classifying disorders, classifications within MILESTONE are based on either DSM or ICD classifications.

**Table S2**

*Measures*

| **Construct** | **Informant (method)** | **Time-point** | **Instruments** | **Description** | **Psychometrics** | **Scoring** |
| --- | --- | --- | --- | --- | --- | --- |
| *Socio-Demographic characteristics* | | | | | | |
| Parental level of education | PC (I) | T1 | Socio-demographic interview (PC-version) | Highest level of PC education of either parent (‘What is your highest completed level of education’). | The socio-demographic interview was largely based on the Client Sociodemographic and Service Receipt Inventory EU version (CSSRI-EU; Chisholm et al., 2000). Psychometric properties of CSSRI-EU for assessing socio-demographic variables are not available, but the instrument has been validated in a large European study on mental health (EPSILON; Chisholm et al., 2000). | Level of PC education was categorized as (1) primary or secondary/vocational or (2) university. If information on the level of education of both PCs was available, we used the level of the PC with the highest educational level. |
| *General factors associated with a recommendation to continue treatment* | | | | | | |
| Clinician-rated severity of psychopathology | CL (I) | T1 | Clinical Global Impression – Severity scale (CGI-S; Guy, 1976) | Clinician rated severity of psychopathology over the last week relative to other patients with similar problems. | The CGI-S is extensively used in psychiatric research (Pinna et al., 2015). | Single score measuring severity on a 7-point scale from ‘not at all ill’ (score = 1) to ‘among the most extremely ill’ (score = 7). |
| Psychotic experiences | YP (OQ) | T1 | Development and Well-Being Assessment (DAWBA; Goodman, Ford, Richards, Gatward, & Meltzer, 2000) | DAWBA assesses a range of psychiatric diagnoses through structured sections of the online questionnaire, among which psychotic experiences (to identify whether the young person has (had) psychosis). The open sections of the DAWBA were omitted to limit the burden on the participants and to standardize the classification procedure. | The DAWBA psychotic experiences section proved valuable as a screening tool in the youth general population (it has not yet been validated in a clinical sample) (Gundersen et al., 2019). | Respondents indicated whether the young person experienced a range of psychotic experiences, with response options ‘no’, ‘a little’, and ‘a lot’. The total number of a total of 10 experiences the young person experienced (either a little or a lot) was calculated. Because the distribution of the number of experiences was zero-inflated, the variable was dichotomized. Having no or 1 psychotic experience(s) was coded as ‘0’, having had 2 or more experiences was categorized as 1. |
|  |  |  |  |  |  |  |
| Everyday functional skills | PC (OQ) | T1 | Specific Levels of Functioning (SLOF; Schneider & Struening, 1983) | Assesses YP’s everyday functional skills. It “emphasizing patient's current functioning and observable behaviour, as opposed to inferred mental or emotional states” (Rocca et al., 2018). | The SLOF domains have acceptable internal consistencies (except for a Cronbach’s alpha of .55 for physical functioning) and good concurrent validity (Mucci et al., 2014). | Average everyday functional skill-scores ranged from 1 to 5 on 6 domains: physical functioning, personal care, interpersonal relationships, social acceptability, activities and work skills, with higher scores indicating more everyday functional skills. A total score was computed to reflect overall everyday functional skills. |
| Psychotropic medication use | YP (OQ) | T1 | CSSRI-EU (amended for use in a psychiatric setting)(Chisholm et al., 2000) | Assesses psychotropic medication use over the last 6 months. |  | Young people provided information on medication use in open text fields. |
| Medical history (length of CAMHS use) | YP (I) | T1 | Socio-demographic interview  (YP-version) | Length of CAMHS use (‘How long have you been receiving care at CAMHS or a related service for mental health, behavioural and/or emotional problems?’ ) was assessed in the socio-demographic interview. | The socio-demographic interview was largely based on the CSSRI-EU, which was found to be effective in tracing patterns of service use in an international population and made comparisons between different countries possible (Chisholm et al., 2000). | Length of CAMHS use was categorized as (1) less than one year, (2) one to five years, or (3) more than five years. |
| Availability of appropriate AMHS | CL (OQ) | T1 | Transition Readiness and Appropriateness Measure (TRAM), developed by the MILESTONE consortium (Santosh et al., 2020) | Availability of appropriate AMHS was assessed with the item “I am confident that there is a local AMHS service with the skills/resources to treat the young person's condition/s.” | The TRAM has been established to be a reliable instrument for assessing transition readiness and appropriateness (Santosh et al., 2020). | The responses were scored as ‘strongly agree’ (2) to ‘strongly disagree’ (-2). |
| Need for ongoing treatment | YP, PC (OQ) | T1 | TRAM | Need for ongoing treatment was assessed with the following item: “does the young person require on-going treatment to control their symptoms?” | See psychometric properties of the TRAM reported for ‘availability of appropriate AMHS’. | Response categories were yes/no |
| *Risk factors for persistent depression* | | | | | | |
| Suicidal thoughts and behaviours | YP (OQ) | T1 | Suicidal thoughts and behaviours were assessed with the TRAM | Suicidal thoughts & behaviours was assessed with the item “I have suicidal thoughts, wish I was dead, imagine how I would kill myself, and/or have attempted to end my own life.” | The TRAM has been established to be a reliable instrument for assessing transition readiness and appropriateness. The ‘suicidal thoughts’ item had high ‘risk’-factor loading score, indicating its relevance to the preparedness of transition (Santosh et al., 2020). | No ‘suicidal thoughts and behaviours’ was scored when the respondent indicated the young person had ‘not experienced’ or ‘rarely experienced’ suicidal thoughts and behaviours. If a response of ‘sometimes’, ‘often’, ‘most of the time’, or ‘all of the time’ was given to the suicidal-thoughts-item, the variable ‘suicidal thoughts and behaviours’ was scored as ‘yes’. |
| History of suicide attempts | YP (I) | T1 | Socio-demographic interview  (YP-version) | History of suicide attempts (‘Have you ever tried to kill yourself?  Have you ever attempted suicide?’) was assessed in the socio-demographic interview. | The socio-demographic interview was largely based on the CSSRI-EU. | Response categories were yes/no |
| Family dysfunction | YP, PC, CL (I) | T1 | The Health of the Nation Outcome Scale for Children and Adolescents (HoNOSCA) (Gowers et al., 1999) | Assesses YP’s health and need for care in the last 2 weeks. In the MILESTONE study, the HoNOSCA is rated by trained research assistants, based on the ‘mental health’-interview with the YP, PCs, the CL and/or medical records.  The HoNOSCA domain ‘Family life and relationships’, was used specifically to assess family dysfunction in the past two weeks. | Good interrater reliability cross-nationally (Hanssen-Bauer et al., 2007), face validity and sensitivity to change in clinical use (Gowers et al., 1999) in adolescent CAMHS patients specifically. Within MILESTONE, research assistants were trained and regular meetings were held to discuss scoring issues and to improve scoring reliability. | The responses on the ‘Family life and relationships’ domain were described as follows: ‘No problems during the period rated’ (0), ‘Slight or transient problems’ (1), ‘Mild but definite problem e.g. some episodes of neglect or hostility or enmeshment or overprotection’ (2), ‘Moderate problems e.g. neglect, abuse, hostility. Problems associated with family/carer breakdown or reorganization’ (3) and; ‘Serious problems with child feeling or being victimized, abused or seriously neglected by family or carer’ (4). |
| Problems with peer-relationships | YP, PC, CL (I) | T1 | HoNOSCA | The HoNOSCA domain ‘Problems with peer relationships’, was used specifically to assess problems with peer relationships in the past two weeks. | See ‘Family dysfunction’ for psychometrics of the HoNOSCA. | The responses on the ‘Problems with peer relationships’ domain were described as follows: ‘No significant problems during the period rated’ (0), ‘Either transient or slight problems, occasional social withdrawal’ (1), ‘Mild but definite problems in making or sustaining peer relationships. Problems causing distress due to social withdrawal, over intrusiveness, rejection or being bullied’ (2), ‘Moderate problems due to active or passive withdrawal from social relationships, over intrusiveness and/or to relationships that provide little or no comfort or support: e.g. as a result of being severely bullied.’ (3) and; ‘Severe social isolation with no friends due to inability to communicate socially and/or withdrawal from social relationships’ (4). |
| Bullying | YP (OQ) | T1 | Adapted from Retrospective Bullying and Friendship Interview Schedule (Wolke & Sapouna, 2008; Zwierzynska, Wolke, & Lereya, 2013) | Assesses the YP’s experiences with bullying in different settings (school, at home, college) | The Retrospective Bullying and Friendship Interview Schedule has previously been used in various populations and was found to be predictive of mental health (Wolke & Sapouna, 2008; Zwierzynska et al., 2013) | Bullying experiences were classified as being a victim of bullying (victim) or not (no victim). |
| Parental psychopathology | PC (I) | T1 | Socio-demographic interview (PC-version) | (History of) Psychopathology in biological parents (“Were you ever examined or treated for mental, developmental, language, speech or learning problems?”) was assessed in the socio-demographic interview. | The socio-demographic interview was largely based on the CSSRI-EU. | Psychopathology in biological parents was categorized as (1) psychopathology in one or both biological parents or (2) no psychopathology. The response was set to missing if the respondent was not a biological parent. |
| Somatic comorbidity | YP (I) | T1 | Socio-demographic interview  (YP-version) | Somatic comorbidity (“Do you have, or have you had, any physical conditions?”) was assessed in the socio-demographic interview | The socio-demographic interview was largely based on the CSSRI-EU (see psychometrics for ‘socio-demographic characteristics’). | Somatic comorbidity was categorized as yes/no. |
| Psychiatric comorbidity | CL (I) | T1 | Clinical classifications | Official clinical classifications registered in the medical records (or, if no official diagnosis was registered: the preliminary/working diagnosis registered). | The classification was based on the Diagnostic and Statistical Manual of Mental Disorders, version IV or 5 and the International Classification of Diseases, version 10. | Psychiatric comorbidity was categorized as yes/no clinical classification of anxiety-, personality- or substance use disorder. |
| *Note*. YP = young person; PC = parent; CL = clinician: I = interview; OQ = online questionnaire | | | | | | |

**Table S3**

*Factors associated with a continuity recommendation for young people with self-reported depressive problems (descriptives and model summary)*

|  | Characteristics  (original non-imputed data) | | | OR [95% CI]  (Model summary on imputed data)* | |  |
| --- | --- | --- | --- | --- | --- | --- |
|  |  | Discontinuity recommendation  (*n* = 33) | Continuity recommendation  (*n* = 115) | Model with general factors | Model expanded with risk factors for persistent depression |  |
| ***General factors associated with a recommendation to continue treatment*** | | | |  |  |  |
| Clinician-rated severity of psychopathology (mean (SD)) |  | 2.44 (0.91) | 4.36 (1.13) | **1.62 [1.12 – 2.34]** | **1.62 [ 1.11 – 2.38]** |  |
| Psychotic experiences (% (*n*)) |  |  |  |  |  |  |
| 0 or 1 experience(s) |  | 30.3 (10) | 40.9 (47) | ref |  |  |
| 2 - 16 experiences |  | 51.5 (17) | 41.7 (48) | 0.51 [0.21 – 1.24] | 0.45 [0.17 – 1.16] |  |
| missing |  | 18.2 (6) | 17.4 (20) |  |  |  |
| Everyday functional skills (mean (SD)) |  | 4.26 (0.36) | 4.18 (0.50) | 0.45 [0.16 – 1.27] | 0.46 [ 0.15 – 1.43] |  |
| Self-reported need for continued  treatment (% (*n*)) |  |  |  |  |  |  |
| No |  | 21.2 (7) | 15.7 (18) | ref |  |  |
| Yes |  | 78.8 (26) | 84.3 (97) | 0.65 [ 0.24 – 1.78] | 0.67 [ 0.23 – 1.92] |  |
| Parent-reported need for continued  treatment (% (*n*)) |  |  |  |  |  |  |
| No |  | 18.2 (6) | 5.2 (6) | ref |  |  |
| Yes |  | 57.6 (19) | 63.5 (73) | 2.54 [0.66 – 9.73] | 2.47 [0.62 – 9.84] |  |
| missing |  | 24.2 (8) | 31.3 (36) |  |  |  |
| ***Risk factors for persistent depression*** | | | |  |  |  |
| Suicidal thoughts and behaviours (% (*n*)) |  |  |  |  |  |  |
| None |  | 45.5 (15) | 34.8 (40) |  |  |  |
| Suicidal thoughts and behaviours |  | 54.5 (18) | 65.2 (75) |  | 0.99 [ 0.43 – 2.29] |  |
| Lifetime suicide attempt (% (*n*)) |  |  |  |  |  |  |
| No |  | 51.5 (17) | 43.5 (50) |  |  |  |
| Yes |  | 48.5 (16) | 51.3 (59) |  | 1.55 [ 0.63 – 3.83] |  |
| missing |  |  | 5.2 (6) |  |  |  |
| Peer Relationship Problems (mean (SD)) |  | 1.00 (1.17) | 1.86 (1.21) |  | 1.05 [0.73 – 1.50] |  |
| Family Dysfunction (mean (SD)) |  | 0.88 (0.93) | 1.82 (1.19) |  | 1.04 [0.69 – 1.56] |  |
| Parental psychopathology (% (*n*)) |  |  |  |  |  |  |
| No psychopathology |  | 33.3 (11) | 35.7 (41) |  |  |  |
| Psychopathology in one or  both biological parents |  | 36.4 (12) | 29.6 (34) |  | 1.44 [ 0.53 – 3.96] |  |
| missing |  | 30.3 (10) | 34.8 (40) |  |  |  |
| Victim of bullying (% (*n*)) |  |  |  |  |  |  |
| No |  | 27.3 (9) | 27.8 (32) |  |  |  |
| Yes |  | 72.7 (24) | 71.3 (82) |  | 0.71 [0.28 – 1.76] |  |
| missing |  |  | 0.9 (1) |  |  |  |
| Somatic Comorbidity^1^ (% (*n*)) |  | 24.2 (8) | 15.7 (18) |  | 1.18 [0.42 – 3.30] |  |
| Psychiatric Comorbidity^2^ (% (*n*)) |  | 42.4 (14) | 31.3 (36) |  | 0.86 [0.36 – 2.05] |  |
|  |  | Optimism Slope | | 0.34 | 0.44 |  |
|  |  | Corrected C-Statistic | | 0.80 | 0.79 |  |

*Note.* Penalized logistic regression models were fitted, displaying odds of ‘continuity recommendation’ versus ‘discontinuity recommendation’ as the reference group. Gender, parental education level and country were added as covariates. **n* changes per imputed dataset. YP = young person, PC = parent/carer. 1 = presence of serious somatic problems such as heart diseases and diabetes, 2 = presence of classifications of anxiety-, personality- and/or substance use disorders.

**Table S4**

*Factors associated with an AMHS recommendation for young people classified with unipolar depressive disorder (descriptives and model summary)*

|  | Characteristics  (original non-imputed data) | | | OR  (Model summary on imputed data)* | |
| --- | --- | --- | --- | --- | --- |
|  |  | CAMHS recommendation  (*n* = 58) | AMHS recommendation  (*n* = 39) | Model with general factors | Model expanded with persistent depression related factors |
| ***General factors associated with a recommendation to continue treatment*** | | | |  |  |
| Psychotropic medication use (% (*n*)) |  |  |  |  |  |
| No |  | 37.9 (22) | 12.8 (5) | *ref* | *ref* |
| Yes |  | 55.2 (32) | 69.2 (27) | 2.95 [0.93 – 9.34] | 3.10 [0.91 – 10.59] |
| missing |  | 6.9 (4) | 17.9 (7) |  |  |
| Length of CAMHS use (% (*n*)) |  |  |  |  |  |
| < 1 yr |  | 32.8 (19) | 28.2 (11) | *ref* | *ref* |
| 1-5yrs |  | 46.6 (27) | 46.2 (18) | 0.90 [0.31 – 2.57] | 0.82 [0.27 –2.48] |
| >5yrs |  | 17.2 (10) | 20.5 (8) | 1.83 [0.51 – 6.55] | 1.77 [0.45 – 6.92] |
| missing |  | 3.4 (2) | 5.1 (2) |  |  |
| Availability of appropriate AMHS (mean (SD)) |  | 0.53 (0.99) | 0.54 (0.91) | 1.16 [0.72 – 1.86] | 1.21 [0.72 – 2.02] |
| Parent-reported need for continued treatment (% (*n*)) |  |  |  |  |  |
| No |  | 6.9 (4) | 5.1 (2) | *ref* | *ref* |
| Yes |  | 63.8 (37) | 71.8 (28) | 0.62 [0.12 – 3.22] | 0.67 [0.12 – 3.94] |
| missing |  | 29.3 (17) | 23.1 (9) |  |  |
| ***Risk factors for persistent depression*** | | | |  |  |
| Clinician-rated severity of psychopathology (mean (SD)) |  | 4.29 (1.11) | 3.82 (1.10) |  | 0.82 [0.51 – 1.30] |
| Suicidal thoughts and behaviours (% (*n*)) |  |  |  |  |  |
| None |  | 51.7 (30) | 48.7 (19) |  | *ref* |
| Suicidal thoughts and behaviours |  | 44.8 (26) | 43.6 (17) |  | 1.37 [0.49 – 3.87] |
| Missing |  | 3.4 (2) | 7.7 (3) |  |  |
| Lifetime suicide attempt (% (*n*)) |  |  |  |  |  |
| No |  | 50.0 (29) | 51.3 (20) |  | *ref* |
| Yes |  | 46.6 (27) | 38.5 (15) |  | 1.02 [0.38 – 2.72] |
| missing |  | 3.4 (2) | 10.3 (4) |  |  |
| Peer Relationship Problems (mean (SD)) |  | 1.57 (1.17) | 1.72 (1.19) |  | 1.14 [0.74 – 1.77] |
| Family Dysfunction (mean (SD)) |  | 1.59 (1.14) | 1.56 (1.07) |  | 1.40 [0.87 – 2.24] |
| Parental psychopathology (% (*n*)) |  |  |  |  |  |
| No psychopathology |  | 37.9 (22) | 41.0 (16) |  | *ref* |
| Psychopathology in one or  both biological parents |  | 31.0 (18) | 30.8 (12) |  | 0.74 [0.23 – 2.32] |
| missing |  | 31.0 (18) | 28.2 (11) |  |  |
| Victim of bullying at school (% (*n*)) |  |  |  |  |  |
| No |  | 24.1 (14) | 28.2 (11) |  | *ref* |
| Yes |  | 70.7 (41) | 59.0 (23) |  | 0.73 [0.24 – 2.23] |
| missing |  | 5.2 (3) | 12.8 (5) |  |  |
| Everyday functional skills (mean (SD)) |  | 4.36 (0.42) | 4.21 (0.53) |  | 1.02 [0.28 – 3.76] |
| Somatic Comorbidity^1^ (% (*n*)) |  | 15.5 (9) | 15.4 (6) |  | 1.09 [0.29 – 4.12] |
| Psychiatric Comorbidity^2^ (% (*n*)) |  | 32.8 (19) | 33.3 (13) |  | 1.28 [0.45 – 3.62] |
|  |  | Optimism Slope | | 0.56 | 0.74 |
|  |  | Corrected C-Statistic | | 0.76 | 0.74 |

*Note.* Penalized logistic regression models were fitted, displaying odds of ‘continuity recommendation’ versus ‘discontinuity recommendation’ as the reference group. Gender, parental education level and country were added as covariates. **n* changes per imputed dataset. YP = young person, PC = parent/carer. 1 = presence of serious somatic problems such as heart diseases and diabetes, 2 = presence of classifications of anxiety-, personality- and/or substance use disorders.

**Table S5**

*Factors associated with an AMHS recommendation for young people with self-reported depressive problems (descriptives and model summary)*

|  | Characteristics  (original non-imputed data) | | | OR  (Model summary on imputed data)* | |
| --- | --- | --- | --- | --- | --- |
|  |  | CAMHS recommendation  (*n* = 54) | AMHS recommendation  (*n* = 41) | Model with general factors | Model expanded with risk factors for persistent depression |
| ***General factors associated with a recommendation to continue treatment*** | | | |  |  |
| Psychotropic medication use (% (*n*)) |  |  |  |  |  |
| No |  | 38.9 (21) | 17.1 (7) |  |  |
| Yes |  | 55.6 (30) | 75.6 (31) | 3.29 [1.13 – 9.59] | 3.51 [1.07 – 11.47] |
| missing |  | 5.6 (3) | 7.3 (3) |  |  |
| Length of CAMHS use (% (*n*)) |  |  |  |  |  |
| < 1 yr |  | 38.9 (21) | 17.1 (7) |  |  |
| 1-5yrs |  | 42.6 (23) | 46.3 (19) | 1.64 [0.60 – 4.50] | 1.89 [ 0.63 – 5.72] |
| >5yrs |  | 16.7 (9) | 31.7 (13) | 2.99 [0.88 – 10.11] | 3.31 [0.85 – 12.79] |
| missing |  | 1.9 (1) | 4.9 (2) |  |  |
| Availability of appropriate AMHS (mean (SD)) |  | 0.61 (1.02) | 0.46 (1.12) | 1.04 [0.68 – 1.60] | 1.10 [0.69 – 1.75] |
| Parent-reported need for continued  treatment (% (*n*)) |  |  |  |  |  |
| No |  | 3.7 (2) | 7.3 (3) |  |  |
| Yes |  | 57.4 (31) | 70.7 (29) | 0.61 [0.12 – 3.09] | 0.55 [0.10 – 3.14] |
| missing |  | 38.9 (21) | 22.0 (9) |  |  |
| ***Risk factors for persistent depression*** | | | |  |  |
| Clinician-rated severity of psychopathology (mean (SD)) |  | 4.40 (1.20) | 4.33 (0.94) |  | 1.15 [0.72 – 1.84] |
| Suicidal thoughts and behaviours (% (*n*)) |  |  |  |  |  |
| None |  | 37.0 (20) | 36.6 (15) |  |  |
| Suicidal thoughts and behaviours |  | 63.0 (34) | 63.4 (26) |  | 1.09 [0.37 – 3.24] |
| Lifetime suicide attempt (% (*n*)) |  |  |  |  |  |
| No |  | 37.0 (20) | 48.8 (20) |  |  |
| Yes |  | 57.4 (31) | 46.3 (19) |  | 0.61 [0.22 – 1.74] |
| missing |  | 5.6 (3) | 4.9 (2) |  |  |
| Peer Relationship Problems (mean (SD)) |  | 1.85 (1.14) | 1.90 (1.30) |  | 1.06 [0.68 – 1.63] |
| Family Dysfunction (mean (SD)) |  | 2.02 (1.12) | 1.54 (1.12) |  | 0.94 [0.60 – 1.47] |
| Parental psychopathology (% (*n*)) |  |  |  |  |  |
| No psychopathology |  | 31.5 (17) | 36.6 (15) |  |  |
| Psychopathology in one or  both biological parents |  | 31.5 (17) | 29.3 (12) |  | 0.63 [0.21 – 1.94] |
| missing |  | 37.0 (20) | 34.1 (14) |  |  |
| Victim of bullying (% (*n*)) |  |  |  |  |  |
| No |  | 33.3 (18) | 26.8 (11) |  |  |
| Yes |  | 66.7 (36) | 70.7 (29) |  | 1.66 [0.58 – 4.80] |
| missing |  |  | 2.4 (1) |  |  |
| Everyday functional skills (mean (SD)) |  | 4.31 (0.44) | 3.98 (0.56) |  | 0.69 [0.21 – 2.25] |
| Somatic Comorbidity^1^ (% (*n*)) |  | 16.7 (9) | 17.1 (7) |  | 1.52 [0.40 – 5.76] |
| Psychiatric Comorbidity^2^ (% (*n*)) |  | 33.3 (18) | 29.3 (12) |  | 1.28 [0.45 – 3.69] |
|  |  | Optimism Slope | | 0.53 | 0.71 |
|  |  | Corrected C-Statistic | | 0.74 | 0.73 |

*Note.* Penalized logistic regression models were fitted, displaying odds of ‘continuity recommendation’ versus ‘discontinuity recommendation’ as the reference group. Gender, parental education level and country were added as covariates. **n* changes per imputed dataset. YP = young person, PC = parent/carer. 1 = presence of serious somatic problems such as heart diseases and diabetes, 2 = presence of classifications of anxiety-, personality- and/or substance use disorders.


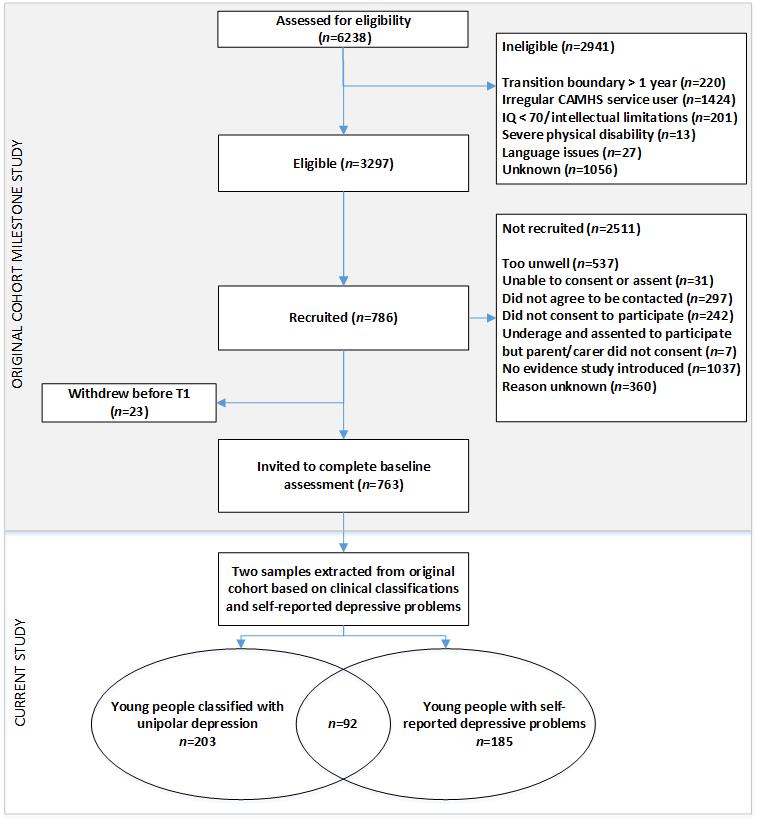


**Figure S1**

*CONSORT Flow Diagram of participants.*

*Note.* CAMHS databases were scrutinised by CAMHS personnel to identify young people approaching the upper age limit of their CAMHS and meeting the inclusion criteria. ). Clinicians introduced the study and reported back to research assistants whether or not young people could be recruited. Due to local privacy laws only limited information could be collected this procedure. Sometimes CAMHS personnel did not provide information on why a young person was not eligible (i.e. Unknown), clinicians indicated that their patient was too unwell to participate in a study at all (i.e. too unwell) or clinicians they did not provide a reason their patient could not be recruited (i.e. reason unknown) or they did not report back at all (i.e. no evidence study introduced).


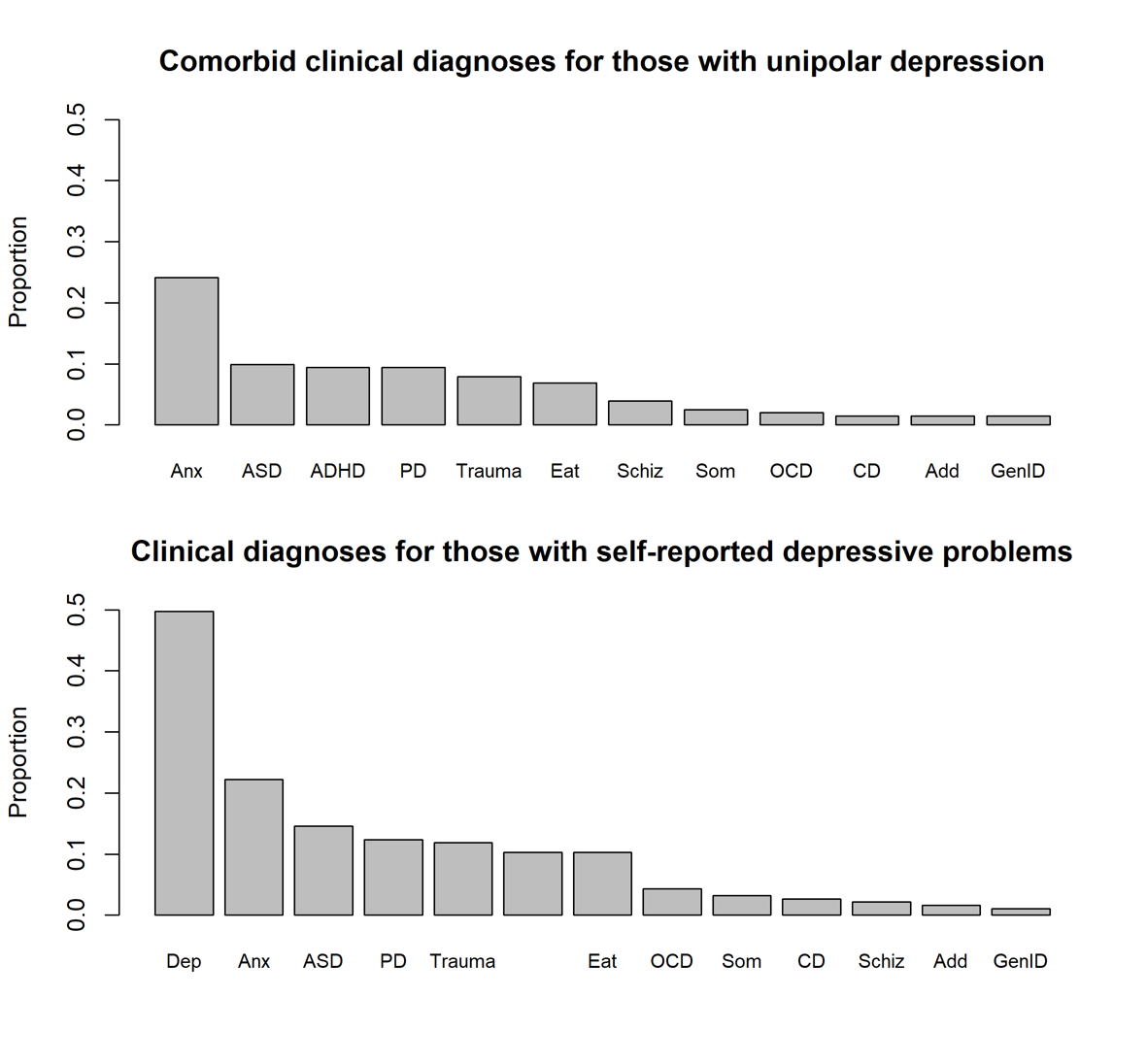


**Figure S2**

*Clinical classifications for young people classified with unipolar depressive disorder (N=203) and young people with self-reported depressive problems (N=185)*

**References**

Chisholm, D., Knapp, M. R. J., Knudsen, H. C., Amaddeo, F., Gaite, L., Van Wijngaarden, B., et al. (2000). Client socio-demographic and service receipt inventory–European version: Development of an instrument for international research: EPSILON study 5. *The British Journal of Psychiatry, 177*(S39), s28-s33.

Goodman, R., Ford, T., Richards, H., Gatward, R., & Meltzer, H. (2000). The development and well-being assessment: Description and initial validation of an integrated assessment of child and adolescent psychopathology. *The Journal of Child Psychology and Psychiatry and Allied Disciplines, 41*(5), 645-655.

Gowers, S. G., Harrington, R. C., Whitton, A., Lelliott, P., Beevor, A., Wing, J., et al. (1999). Brief scale for measuring the outcomes of emotional and behavioural disorders in children: Health of the nation outcome scales for children and adolescents (HoNOSCA). *The British Journal of Psychiatry, 174*(5), 413-416.

Gundersen, S. V., Goodman, R., Clemmensen, L., Rimvall, M. K., Munkholm, A., Rask, C. U., et al. (2019). Concordance of child self‐reported psychotic experiences with interview‐and observer‐based psychotic experiences. *Early Intervention in Psychiatry, 13*(3), 619-626.

Guy, W. (1976). ECDEU assessment manual for psychopharmacology. *US Department of Health, and Welfare,* , 534-537.

Hanssen-Bauer, K., Gowers, S., Aalen, O. O., Bilenberg, N., Brann, P., Garralda, E., et al. (2007). Cross-national reliability of clinician-rated outcome measures in child and adolescent mental health services. *Administration and Policy in Mental Health and Mental Health Services Research, 34*(6), 513-518.

Mucci, A., Rucci, P., Rocca, P., Bucci, P., Gibertoni, D., Merlotti, E., et al. (2014). The specific level of functioning scale: Construct validity, internal consistency and factor structure in a large italian sample of people with schizophrenia living in the community. *Schizophrenia Research, 159*(1), 144-150.

Pinna, F., Deriu, L., Diana, E., Perra, V., Randaccio, R. P., Sanna, L., et al. (2015). Clinical global impression-severity score as a reliable measure for routine evaluation of remission in schizophrenia and schizoaffective disorders. *Annals of General Psychiatry, 14*(1), 1-8.

Rocca, P., Galderisi, S., Rossi, A., Bertolino, A., Rucci, P., Gibertoni, D., et al. (2018). Disorganization and real-world functioning in schizophrenia: Results from the multicenter study of the italian network for research on psychoses. *Schizophrenia Research, 201*, 105-112.

Santosh, P., Singh, J., Adams, L., Mastroianni, M., Heaney, N., Lievesley, K., et al. (2020). Validation of the transition readiness and appropriateness measure (TRAM) for the managing the link and strengthening transition from child to adult mental healthcare in europe (MILESTONE) study. *BMJ Open, 10*(6), e033324.

Schneider, L. C., & Struening, E. L. (1983). SLOF: A behavioral rating scale for assessing the mentally ill. *Social Work Research and Abstracts, , 19.* (3) pp. 9-21.

Wolke, D., & Sapouna, M. (2008). Big men feeling small: Childhood bullying experience, muscle dysmorphia and other mental health problems in bodybuilders. *Psychology of Sport and Exercise, 9*(5), 595-604.

Zwierzynska, K., Wolke, D., & Lereya, T. S. (2013). Peer victimization in childhood and internalizing problems in adolescence: A prospective longitudinal study. *Journal of Abnormal Child Psychology, 41*(2), 309-323.
